# The Management of Obstructive Sleep Apnea in Primary Care

**DOI:** 10.7759/cureus.26805

**Published:** 2022-07-13

**Authors:** Gokul Paidi, Anju Beesetty, Marie Jean, Farrah P Aziz Greye, Taha Siyam, Maria F Fleming, Joshua Nealy, Lisa Kop, Ranbir Sandhu

**Affiliations:** 1 Pulmonary and Critical Care Medicine, Arizona Lung Sleep And Valley Fever Institute, Surprise, USA; 2 Obstetrics and Gynecology, Southern Medical University, Guangzhou, CHN; 3 Research, California Institute of Behavioral Neurosciences & Psychology, Fairfield, USA; 4 Internal Medicine, 24/7 Urgent Care, Saint Louis, USA; 5 Family Medicine, Umm Al-Qura University, Mecca, SAU; 6 Medicine, Forum of Artificial Intelligence in Medicine, Miami, USA; 7 Medicine, Universidad Central de Venezuela, Caracas, VEN; 8 General Medicine, Mildred Elley, Pittsfield, USA; 9 Neurology, Zaporizhzhia State Medical University, Zaporizhia, UKR; 10 Urgent Care, San Luis Walk in Clinic, Yuma, USA

**Keywords:** polysomnography, obstructive sleep apnea (osa), continuous positive airway pressure (cpap), epworth sleepiness scale, insomnia

## Abstract

Approximately 30 million Americans suffer from sleep disorders. The incidence of and mortality rates associated with obstructive sleep apnea (OSA) have been increasing in recent years in the United States. OSA is associated with various health problems, including depression and hypertension, and it adversely affects occupational and academic performance. Hence, OSA is a major public health concern. Sleep specialists may be consulted for the evaluation and treatment of OSA. Continuous positive airway pressure (CPAP) is the mainstay of OSA treatment. The role of primary care physicians in such a scenario becomes vital, especially for choosing the most suitable approach for each patient, treating comorbidities and risk factors, and, if needed, referring them to sleep specialists for further management. In addition to medical management, primary care physicians serve as the main patient educator on this particular health condition.

## Introduction and background

**Figure 1:**
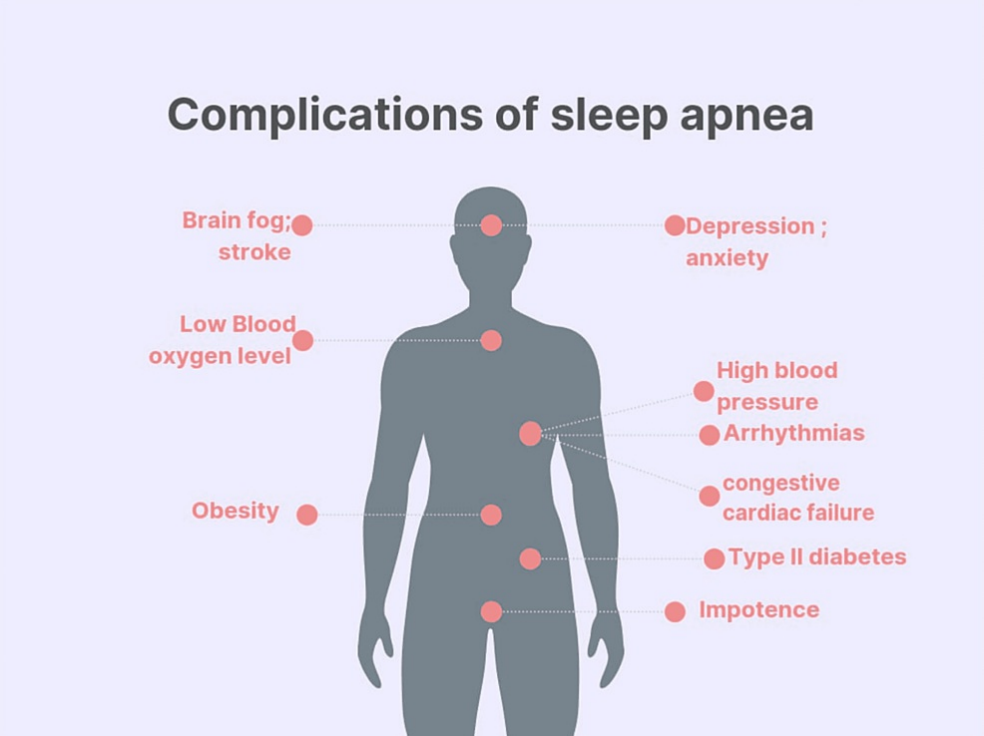
Complications of obstructive sleep apnea Image credit: Gokul Paidi

Obstructive sleep apnea (OSA) is a very common condition, affecting more than 10% of the adult population, and its prevalence increases with age [1]. OSA is caused by the intermittent collapse of the upper airways while sleeping; this leads to transient asphyxia and thus hypoxia. OSA remains a vital public health concern and is associated with the onset or worsening of hypertension, and other health problems, including cardiovascular disease and stroke, and overall poor quality of life [2]. OSA affects work performance and can result in road traffic accidents [3]. Of note, 70% of patients with resistant hypertension suffer from undiagnosed OSA. The use of continuous positive airway pressure (CPAP) can help control blood pressure [4]. Moreover, high sleep quality can improve memory, thinking, mood, alertness, energy level, and physical performance. However, only about 10% of patients with OSA are identified. The inadequate diagnosis and treatment of OSA directly affect public health due to the economic burden of untreated OSA in the long term [5]. Complications associated with OSA are presented in Figure 1.

Increased demand for specialized sleep units for the purpose of sleep service provision along with increasing wait lists for consultations for these sleep provisions have been observed. Chronicity and chronic health conditions of this disease, higher prevalence, and long-term consequences make the management of this disease imperative. Similar to other prevalent chronic diseases, the comprehensive management and care of OSA should include other clinical settings as well. In primary care settings, a novel model for the management of OSA has been proposed [6]. Studies conducted on patients having a high pre-test probability of OSA have shown that this model of management is achievable in primary care centers. This demonstrates comparable effectiveness with the sleep unit models [6]. Primary care is the most cost-effective and thorough setting for treating individuals with suspected OSA. This will have an impact on the utilization of diagnostic testing methods and different treatment plans that are yet to be explored. The aim of this article is to explore the diagnosis and management of OSA and its complications in primary healthcare settings.

## Review

Approaches for the diagnosis of OSA

**Figure 2:**
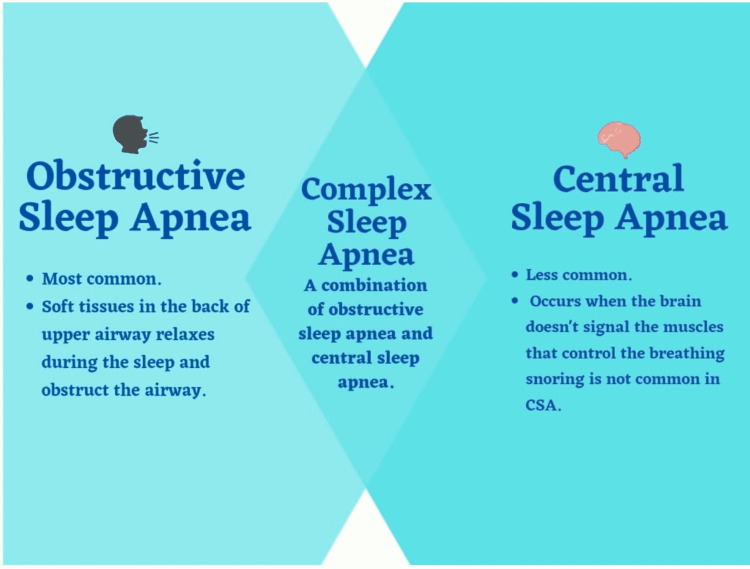
Types of sleep apnea Image credit: Gokul Paidi

Sleep Apnea can be classified into three types: 1. obstructive, 2. central, and 3. complex (Figure 2).

During the diagnosis of OSA, ruling out other causative factors that may cause sleep disturbances is essential [7]. An airway examination should be performed by determining patency and neck circumference measurements. The thyroid-stimulating hormone level should be measured to determine whether obstruction is caused by an enlarged thyroid gland [8]. Primary care physicians must ask questions on sleep hygiene, sleep quality and duration, comorbid illnesses, drug usage, stressful events in life, work and daily schedule, use of digital devices, bedroom settings, alcohol and caffeine intake, and other medical and mental health conditions interfering with sleep [7]. Diagnosis of OSA in older patients can be challenging for primary care physicians because changes related to age can result in changes in the sleep cycle [7-8]. Moreover, older individuals usually receive medication that can cause drowsiness or restlessness as side effects. Thus, primary care physicians should examine if excessive sleepiness is a symptom of other health conditions or a side effect of prescribed drugs [9].

The STOP-BANG questionnaire is used to determine the risk of sleep apnea (Table 1), and excessive sleepiness is screened using the Epworth Sleepiness Scale (ESS; Table 2) [10].

**Table 1:** STOP-BANG questionnaire* *[11] Interpretation: low risk: 0-3, intermediate risk: 4-5, high risk: 6-8

Please answer the following question by checking "Yes "or "No" for each one	YES	NO
Snoring (do you snore loudly?)	⭕	⭕
Tiredness (do you often feel tired, fatigued, or sleepy during the daytime?)	⭕	⭕
Observed apnea (has anyone observed that you stop breathing or choke or gasp during your sleep?)	⭕	⭕
High blood pressure (do you have or are you being treated for high blood pressure?)	⭕	⭕
BMI (is your body mass index more than 35 kg per m²?)	⭕	⭕
Age (are you older than 50 years?)	⭕	⭕
Neck circumference [is your neck circumference greater than 40 cm (15.75 inches)?] Gender (are you male?)	⭕	⭕
Total score:		

**Table 2:** Epworth Sleepiness Scale* *[12] Interpretation: 0-10: normal range of sleepiness in healthy adults, 11-14: mild sleepiness, 15-17: moderate sleepiness, 18-24: severe sleepiness

How likely you are to fall asleep in the situations described below					
Situation	Chance of sleeping				
	No		Slight	Moderate	High
Sitting and reading	0		1	2	3
Watching television	0		1	2	3
Sitting inactive in public space	0		1	2	3
Lying down to rest in the afternoon	0		1	2	3
Sitting and talking to someone	0		1	2	3
As a passenger in a car for an hour without a break	0		1	2	3
In a car, while stopped for a few minutes in a traffic	0		1	2	3
Total score:					

Initial diagnosis confirmation

Patients suspected of having OSA should be referred to a sleep specialist for polysomnography (PSG). A sleep specialist’s involvement in the early stage of the diagnosis of sleep apnea enables the use of PSG in accordance with patients’ needs. This will allow for a precise interpretation of results and enable primary care physicians and sleep specialists to work together for treatment planning, implementation, and follow-up [10]. Polysomnogram performed in a sleep laboratory is the gold standard for diagnosing sleep disorders. This test can help obtain comprehensive signals from brain waves, breathing patterns, heart rate, oxygen saturation, and body movement. Sleep-center PSG can be used to diagnose all three types of apnea as well as rapid eye movement sleep disorders, seizure disorders related to sleep (nocturnal seizures), movement disorders related to sleep (periodic limb movement of sleep disorder, parasomnias, and restless leg syndrome), and narcolepsy [13].

Home sleep testing (HST) was initially used in most patients due to cost constraints and patient preference. Technical difficulties related to home sleep study include poor lead placement, maintenance through the night during sleep, and lack of health education. Because HST does not measure brain wave activity and body movements, it cannot be used to diagnose sleep-related seizures and movement disorders. Moreover, HST is ineffective for patients with severe heart or lung disease, preexisting major sleep difficulties, neuromuscular disorders, a history of stroke, and persistent opioid use [10,13]. The difference between a laboratory sleep study and a home sleep study is explained in Table 3.

**Table 3:** Difference between lab and home sleep studies* *[14] CPAP: continuous positive airway pressure

Lab sleep study	Home sleep study
Electrodes to monitor brain waves, heart rate, muscle activity, oxygen saturation, limb movements	Pulse oximeter or similar device
Chest belt that senses and counts breathing	Chest belt
Nasal cannula	Chest sensor
CPAP machine (only for undergoing titration)	Nasal cannula

A threshold of ≥5 scorable respiratory events in an hour is utilized to diagnose OSA. These events can comprise any amalgamation of obstructive apnea, arousal, or hypopnea [15]. Information on the following terminologies (Table 4) is essential to read a sleep study report.

**Table 4:** Sleep study report terminologies* *[16]

Term	Definition
Apnea	Temporary cessation of airflow ≥10 seconds. Can be obstructive, central, and mixed
Hypopnea	A drop in airflow minimum of 30% that lasts for 10 seconds or more and results in at least 4% desaturation
Respiratory effort-related arousal (RERA)	Subtle fluctuation of respiratory airflow. Does not meet the criteria of apnea or hypopnea
Apnea-hypopnea index (AHI)	(Total number of apneas + total number of hypopneas)/sleep hours
Respiratory disturbance index (RDI)	Apneas + hypopneas + RERA

The apnea-hypopnea index (AHI) is the main and most common parameter used to diagnose OSA. The grading of OSA based on AHI is presented in Table 5.

**Table 5:** Grading of AHI* *[16] AHI: apnea-hypopnea index

AHI grading	
None/normal	AHI <5 per hour
Mild	AHI ≥5 per hour
Moderate	AHI ≥15 per hour, but <30 per hour
Severe	AHI ≥30 per hour

The AHI varies with the position of the body; it is higher when a person sleeps in the supine position when the risks of the tongue falling back and obstruction are high. Moreover, the duration of sleep in supine, prone left, and right positions should be determined, and its correlation with SPO_2_ and the snoring intensity should be evaluated.

Management of obstructive sleep apnea

CPAP

CPAP is the gold standard for the management of OSA [17]. CPAP delivers a predetermined level of pressure that maintains the airway open during sleep. The pressure setting ranges from 4 to 30 cmH_2_O. Individuals’ pressure setting is determined by a pulmonologist based on their AHI, sleep position, and respiratory disturbance index (RDI). A low-pressure setting should be initially used for new patients to help them adjust to mask use and pressure. CPAP can considerably improve patients’ quality of life and reduce blood pressure in patients with hypertension [17]. Moreover, CPAP improves lung mechanics by recruiting microatelectasis and increasing the diffusion capacity. The use of CPAP in patients with lung and heart failure improves hemodynamics by reducing preload and afterload and left ventricular transmural pressure. In addition, CPAP has been reported to reduce the need for intubation [18]. Dryness of the nose and throat is the most common side effect of CPAP. However, adjusting the humidifier level may alleviate this problem. Dryness and soreness of the eyes are other side effects caused by air leaks from the mask. The use of an appropriately fitting mask can address this problem. Aerophagia, a condition in which air enters the stomach, is typically caused by high- or low-pressure settings. Aerophagia can be prevented by adjusting the pressure relief setting on CPAP, which allows individuals to adjust air pressure during exhalation, using full face masks to prevent mouth breathing, and sleeping in an upward angle of 30-40° [13,15,17,18,19].

Other Positive Airway Devices

The use of automatic self-adjusting positive airway pressure (APAP) is another option for CPAP management. The lowest possible air pressure is delivered in APAP to maintain an open airway [20]. This strategy may be helpful for individuals who experience difficulty adjusting to the constant pressure of the CPAP device [21]. The bilevel positive airway pressure is another device used to mimic normal breathing and involves the application of low pressure at the time of expiration compared with CPAP [22]. This is an assisted ventilation technique that is more effectively tolerated by some patients with OSA and those who additionally experience awake hypercapnia, hyperventilation syndrome, or chronic obstructive pulmonary disease [23]. Adaptive servo-ventilation (ASV) is another noninvasive ventilatory device used for the treatment of mixed, central, and complex sleep apnea and involves constantly adjusting the pressure based on patients’ breathing patterns during both inspiration and expiration [23,24].

CPAP Monitoring

Patients receiving treatment for OSA should be followed up in the primary care setting. The first phase of management should include the assessment of CPAP acceptability and compliance, involvement of the patient support team, and provision of adequate knowledge about the device. Physicians should discuss problems such as pain or nasal dryness related to the use of the CPAP device [25,26]. Snoring persistence should be measured by considering the perceptions of individuals’ partners and improvement in patients’ quality of life and activities during the daytime. Excessive sleepiness is a key measure to be examined in individuals receiving CPAP treatment [27]. Residual excessive sleepiness can be treated using adjunctive pharmaceutical agents. CPAP use at night for the first few weeks can aid in long-term compliance prediction. CPAP use of <2 hours per night was associated with discontinuation or inconsistent use [28,29]. A study reported a dose-dependent relationship between improvement in daily functioning and sleepiness with the duration of CPAP use at night [30]. Individuals with complete resolution of excessive sleepiness received CPAP treatment every night for ≥1 hour compared with those with residual excessive sleepiness [30].

If patients experience difficulty during exhalation or cannot tolerate CPAP pressure or dry mouth, then alternate devices for positive airway pressure might be more suitable. Periodic desensitization to a CPAP mask may lead to improved compliance. The process of desensitization includes patients wearing a mask for shorter durations during the day while being in a standing or upright position and utilizing low air pressure before increasing the pressure to the necessary level [7]. White noise machines may help maintain compliance in patients whose sleep is disrupted by CPAP. Heated humidification of CPAP air may improve comfort in patients with dryness and nasal congestion [31].

Education should be provided to patients on the suitable maintenance of CPAP devices to ensure that they receive optimal treatment and to increase the device lifespan. CPAP masks should be wiped daily. The humidifier should be thoroughly washed each week. Support and maintenance vary between providers and machines. CPAP machine providers should provide filters and new headgear when needed as well as calibrate the machine for ensuring accurate air pressure when physicians prescribe it. All these services and goods should be covered by the medical insurance of patients [7].

Patients should be followed up every four to five months to examine their compliance. Patients treated with CPAP for the long term should receive annual counseling on comfort, adherence, fit, and any additional requirements regarding the maintenance and use of the equipment. Reassessment of patients should include increasing or decreasing their weight by >10% along with checking for the development of any new comorbidities. Patients with OSA might have questions regarding the use of CPAP in case they experience any upper respiratory tract infection (i.e., influenza). Hence, primary care physicians should reassure them to stay compliant with their usual regimen for CPAP and use nasal saline, nasal sprays, decongestants, and humidification [32,33].

Oral Appliances

The use of oral appliances has emerged as an alternative for patients with OSA who experience difficulty in tolerating CPAP and who have mild sleep apnea [34]. Compared with CPAP, the ease of use may determine individual preference for oral appliance choice and thus may lead to better adherence and health outcomes. These devices include the mandibular advancement device, mouth guard, and tongue retention device. All these devices move the jaw and tongue forward and increase the size of the airway.

Surgery

**Figure 3:**
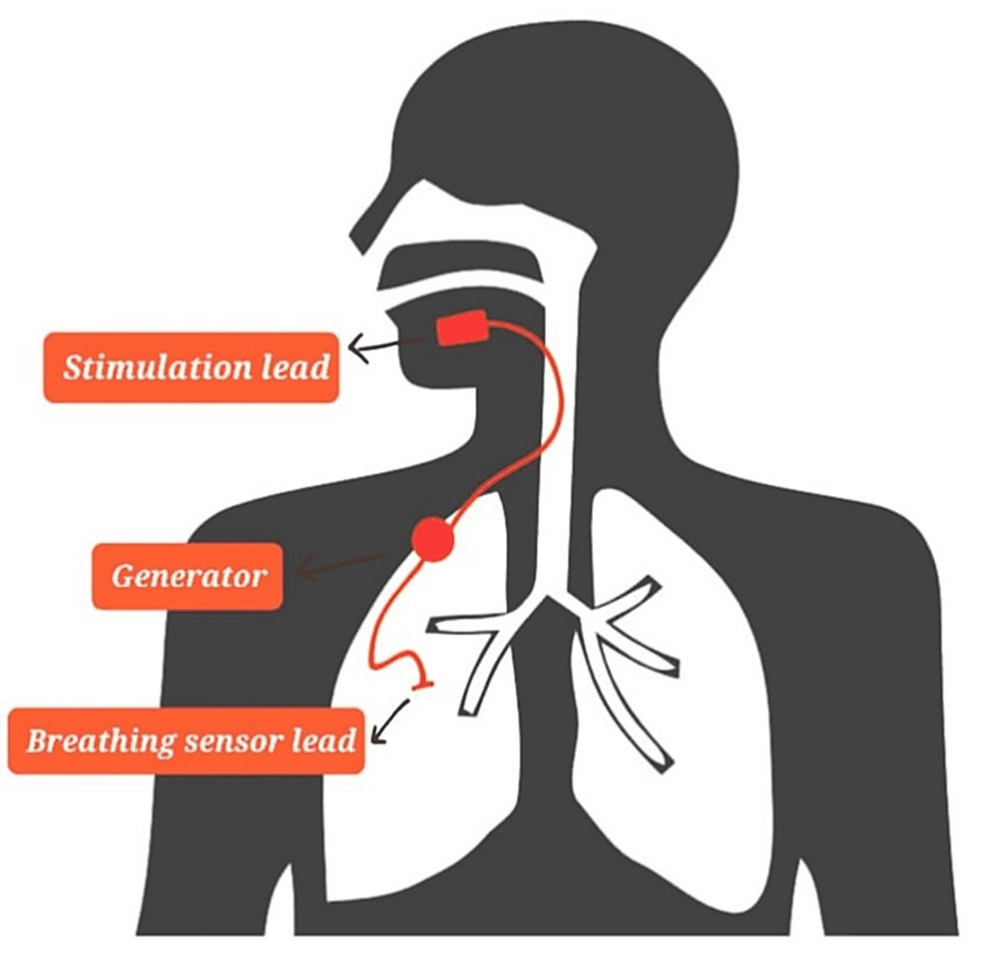
Hypoglossal nerve stimulator Image credit: Gokul Paidi

The hypoglossal nerve stimulator (Figure 3) prevents OSA by electrically stimulating the hypoglossal nerve in a timed manner in correlation with respiration [33].

Other Therapies

Studies have recommended using dilation clips for the nose, maintaining the upright position during sleeping, avoiding sedatives or alcohol, and losing weight while using CPAP, which is the primary therapy [34-36]. Supplemental oxygen may be used as an adjunct for the treatment of patients with OSA with lower oxygen saturation levels. However, supplemental oxygen would not address problems related to comorbidities [35,36].

Evaluation of Residual Excessive Sleepiness

In some patients receiving CPAP treatment, even after adequate sleep oxygen therapy and correction of all other sleep-related parameters, daytime sleepiness, known as excessive residual sleepiness, is observed. In these individuals, any underlying cause of sleep or neurological disorders should be excluded. If the cause cannot be identified, then medication should be started. Modafinil has been reported to significantly improve residual excessive sleepiness in patients with OSA when administered as adjunctive therapy along with CPAP [36,37].

## Conclusions

In primary care settings, most patients presenting with sleep-related problems suffer from undiagnosed OSA. Both OSA and excessive sleepiness related to it can affect the quality of life and cause mortality and morbidity, thereby resulting in a significant economic burden on the healthcare system. The reduction in mortality and morbidity related to OSA requires appropriate treatment. CPAP is the mainstay of therapy for the management of OSA. Appropriate diagnosis at an early stage may help improve associated complications. The role of primary care physicians is vital for the diagnosis of OSA and the initiation of appropriate treatment. Primary care physicians may also address associated complications, such as depression and loss of quality of life. Regular follow-ups are crucial to assess complications associated with OSA management. On the basis of additional symptoms, such as nose dryness or excessive sleepiness, additional therapies may be warranted. Clinicians should support these individuals during the course of the entire treatment and ensure that individuals are provided with appropriate information regarding available options and devices for the management of OSA. The monitoring of patients’ progress is critical for addressing issues related to the treatment of OSA.
